# Supplementary material for: The mitochondrial calcium uniporter of pulmonary type 2 cells determines severity of acute lung injury
Source: Nat Commun. 2022 Oct 3;13:5837. doi: 10.1038/s41467-022-33543-y (PMC9529882; doi:10.1038/s41467-022-33543-y)
Supplement: Supplementary file 1 — Supplementary Information [file 41467_2022_33543_MOESM1_ESM.pdf]

The Mitochondrial Calcium Uniporter of Pulmonary Type 2 Cells Determines Severity of Acute Lung Injury

Mohammad Naimul Islam[1], Galina A. Gusarova[1], Shonit R. Das[1†], Li Li[1], Eiji Monma[1], Murari Anjaneyulu[2], Liberty Mthunzi[1], Sadiqa K. Quadri[1], Edward Owusu-Ansah[2], Sunita Bhattacharya[3] and Jahar Bhattacharya[1,2,4,*]

[1]Department of Medicine, Vagelos College of Physicians and Surgeons, Columbia University, New York, NY-10032

[2]Department of Physiology and Cellular Biophysics, Vagelos College of Physicians and Surgeons, Columbia University, New York, NY-10032

[3]Department of Pediatrics, Vagelos College of Physicians and Surgeons, Columbia University, New York, NY-10032

[4]Lead contact

[†]Deceased

*Correspondence: jb39@cumc.columbia.edu

Supplementary Methods

Supplementary Table 1: List of reagents and resources used

REAGENT or RESOURCE	SOURCE	IDENTIFIER
Experimental model: Organisms/Strains		
Mouse: C57BL/6J	The Jackson Laboratory	JAX: 000664
Mouse: Swiss Webster	Taconic	
Mouse: MCU$^{F/F}$ C57BL/6J	Ref [1]	N/A
Mouse: SPC-rtTA/TetO-Cre	Ref [2]	N/A
Mouse: SPC-ERT2-Cre	The Jackson Laboratories	JAX: 028054
Mouse: FVB/NJ	The Jackson Laboratories	JAX: 001000
Mouse: mCAT$^{F/F}$ C57BL/6J	Ref [3]	N/A
Mouse: Cx43$^{F/F}$ C57BL/6J	The Jackson Laboratories	JAX: 008039
Mouse: PhAM floxed x E2a- Cre	Ref [4]	N/A
Mouse: Drp1$^{F/F}$ C57BL/6J	Ref [5]	N/A
Experimental model: Cell line		
Bone marrow-derived mesenchymal stromal cells	This paper	N/A
3T3-L1 fibroblasts	ATCC	Cat# CL-173
Oligonucleotides		
ON-TARGET plus non-targeting pool	GE Healthcare Dharmacon	D-001810-10-05
Alexa Flour 647-MCU siRNA	Qiagen	SI00966868
Dy547-RISP siRNA	GE Healthcare Dharmacon	J-037755-01
Mouse: β-Actin Forward GGCTGTATTCCCCTCCATCG	PrimerBank	https://pga.mgh.harvard.edu/primerbank/
Mouse: β-Actin Reverse CCAGTTGGTAACAATGCCATGT	PrimerBank	https://pga.mgh.harvard.edu/primerbank/
Mouse: MCU Forward AAAGGAGCCAAAAAGTCACG	Ref [6]	N/A
Mouse: MCU Reverse AACGGCGTGAGTTACAAACA	Ref [6]	N/A
Recombinant DNA		
pMCU-GFP-wt	Ref [6]	N/A
pMCU-GFP-NQ (*pMCUmt*)	Ref [6]	N/A
Human SPC promoter	Ref [7]	N/A
Matrix roGFP	Ref [8]	Addgene: Plasmid# 49437
ChR2-GFP	Ref [9]	Addgene: Plasmid# 15814
K38A Drp1	Ref [10]	Addgene: Plasmid# 45161
Chemicals, media and peptides		
Dulbecco's Modified Eagle Media (DMEM)	Corning	Cat# 10-013-CV
Opti-MEM	GIBCO	Cat# 31985-070
LB Medium	MP Biomedicals	Cat# 3002-031
1X DPBS with Ca^{2+} and Mg^{2+}	Corning	Cat# 21-030-CV
1X DPBS without Ca^{2+} and Mg^{2+}	Corning	Cat# 21-031-CV
Heat-Inactivated Fetal Bovine Serum	GE Healthcare	Cat# SH30071.03HI
Penicillin/Streptomycin	Corning	Cat# 30-002-CI
Carbenicillin	Sigma-Aldrich	Cat# C1389
Trypsin	Sigma-Aldrich	Cat# T4046
Tail buffer	Teknova	Cat# T0525
Proteinase K	Ambion	Cat# AM2546
Natural Protease (Dispase)	Worthington Biochemicals	Cat# LS02104

T25 tissue culture flasks	Corning	Cat # 353109
T75 tissue culture flasks	Corning	Cat # 353136
Lipopolysaccharide (E. coli 0111:B4)	Sigma-Aldrich	Cat# L4130
Ruthenium Red	Calbiochem	Cat# 567450
FCCP	Sigma-Aldrich	Cat# C2920
Rotenone	Sigma-Aldrich	Cat# R-8875
MitoQ	Ref [11]	N/A
Mdivi-1	Sigma-Aldrich	Cat# M0199
DMSO	Fisher Scientific	Cat# BP231-100
Xestospongin C	Abcam	Cat# ab120914
Dithiothreitol (DTT)	Bio-Rad	Cat# 161-0611
Hydrogen peroxide (H2O2)	Sigma-Aldrich	Cat# H1009
Paraformaldehyde	Electron Microscopy Sci.	Cat# 15710
Triton	Sigma-Aldrich	Cat# X100
Halt protease & phosphatase inhibitor cocktail	Thermo Scientific	Cat# 78442
Fluo-4	Molecular Probes	Cat# F14201
Rhod-2	Molecular Probes	Cat# R1245MP
Lysotracker red (LTR)	Molecular Probes	Cat# L7528
Lysotracker green (LTG)	Molecular Probes	Cat# L7526
Tetramethylrhodamine, methyl ester (TMRE)	Molecular Probes	Cat# T668
Calcein AM	Molecular Probes	Cat# C3100MP
Calcein red-orange	Molecular Probes	Cat# C34851
Hoechst 33342	Molecular Probes	Cat# H3570
DCF	Molecular Probes	Cat# D141
BAPTA-AM	Molecular Probes	Cat# B6769
Human surfactant protein B	Ref [12]	N/A
BODIPY	Molecular Probes	Cat# D2183
TO-PRO-3	Molecular Probes	Cat# T3605
DMSO with 20% pluronic acid	Molecular Probes	Cat# P3000MP
Doxycycline	Sigma-Aldrich	Cat# D9891
Tamoxifen	Sigma-Aldrich	Cat# T5648
Corn Oil	Sigma-Aldrich	Cat# C8267
Surfactant (Curosurf® – 80mg/ml)	Chiesi, USA	
Commercially available kits		
Mitochondria isolation kit	Thermo Scientific	Cat# 89801
ATP detection kit	Abcam	Cat# ab83355
Software and Algorithms		
Zeiss AIM	Carl-Zeiss	Version 4.7 SP1
Leica Application Suite	Leica	Advanced Fluorescence
ImageJ	NIH (version 1.53c)	https://imagej.nih.gov/ij/
FIJI	Ref [13]	www.fiji.sc
MetaMorph	Molecular Devices	Version 7.8
FlowJo	TreeStar	Version 10
SigmaPlot	Systat	Version 12.5
Microsoft Excel	Microsoft Corporation	2013

Supplementary Table 2: List of antibodies used

Antibodies	SOURCE	Catalog #	DILUTION
Rabbit anti-MCU	Sigma-Aldrich	Cat#HPA016480	1:1000
Rabbit anti-MCU (D2Z3B)	Cell Signaling Technologies	Cat#14997	1:1000
Rabbit anti-VDAC	Cell Signaling Technologies	Cat#4866	1:1000
Anti-actin	Sigma-Aldrich	Cat# A2066	1:1000
Mouse anti-Drp1 (C-5)	Santa Cruz Biotechnology	Cat# sc-271583	1:1000
Rabbit anti-Drp1, phospho (Ser616)	Cell Signaling Technologies	Cat#34555	1:1000
Mouse anti-HSP60 (H-1)	Santa Cruz Biotechnology	Cat# sc-13115	1:1000
Mouse anti-TOM20 (F-10)	Santa Cruz Biotechnology	Cat# sc-17764	1:1000
Human anti-catalase	Athens Research	Cat# 01-05-030000	1:1000
Catalase	Abcam	Cat# ab88067	1:1000
Cytochrome oxidase (COX) IV (F-8)	Santa Cruz Biotechnology	Cat# sc-376731	1:1000
Mouse anti-GFP (B-2)	Santa Cruz Biotechnology	Cat# sc-9996	1:1000
Mouse anti-GFP (B-2) Alexa Fluor-647	Santa Cruz Biotechnology	Cat# sc-9996 AF647	1:250s
Mouse anti-connexin 43 (Cx43) (F-7)	Santa Cruz Biotechnology	Cat# sc-271837	1:1000
Rabbit anti-connexin 43 (Cx43) (H-150)	Santa Cruz Biotechnology	Cat# sc-9059	1:1000
Mouse anti-Rieske Iron Sulfur Protein (A-5)	Santa Cruz Biotechnology	Cat# sc-271609	1:1000
Mouse anti-SDH (G-10)	Santa Cruz Biotechnology	Cat# sc-271548	1:1000
Mouse anti-ATP γ-synthase (3D5AB1)	Thermo Scientific	Cat# A21351	1:1000
Mouse anti-PINK1	Santa Cruz Biotechnology	Cat# sc-517353	1:1000
Mouse anti-Parkin (PRK8)	Santa Cruz Biotechnology	Cat# sc-32282	1:1000
Mouse anti-CD11b (M1/70)	eBioscience	Cat# 48-0112-80	1:1000
Mouse anti-CD11c (N418)	Biolegend	Cat# 117310	1:1000
Mouse anti-CD45 (30-F11)-pacific blue	Biolegend	Cat# 103126	1:1000
Mouse anti-Ly-6G (RB6-8C5)	Thermo Fisher Scientific	Cat# 12-5931-82	1:1000
Rat anti-CD29 (Ha2/5)	BD Biosciences	Cat# 555005	1:1000
Rat anti-CD44 (OX-49)	BD Biosciences	Cat# 550974	1:1000
Rat anti-CD59 (TH9)	BD Biosciences	Cat# 550976	1:1000
Rat anti-CD71 ((OX-26)	BD Biosciences	Cat# 554890	1:1000
Rat anti-CD90.1 (OX-7)	BD Biosciences	Cat# 554897	1:1000
Rabbit anti-surfactant protein B (H-300)	Santa Cruz Biotechnology	Cat# sc-13978	1:1000
Goat anti-surfactant protein C (M-20)	Santa Cruz Biotechnology	Cat# sc-7706	1:1000
AF 488 goat anti-rabbit IgG	Molecular Probes	Cat# A-11008	1:1000
IRDye® 680RD Goat anti-Mouse IgG	LI-COR Biosciences	Cat# 926-68070	1:10000
IRDye® 800CW Goat anti-Mouse	LI-COR Biosciences	Cat# 926-32210	1:10000
IRDye® 680LT Goat anti-Rabbit IgG	LI-COR Biosciences	Cat# 926-68021	1:10000
IRDye® 800CW Goat anti-Rabbit IgG	LI-COR Biosciences	Cat# 926-32211	1:10000

Supplementary Table 3: Animals strains and LPS doses used

STUDY	DOSE (mg/kg body weight)	STRAIN	LETHALITY IN CONTROLS (%)	ALI SEVERITY
Alveolar physiology and stretch responses	1	SW	0	Nonlethal
$AT2^{CAT+/+}$ physiology	1	Mixed (B6 x FVB)	0	Nonlethal

AT2$^{Drp1-/-}$ physiology	1	Mixed (B6 x FVB)	0	Nonlethal
BM-MCU physiology	1	SW	0	Nonlethal
pMCUwt physiology	1	SW	0	Nonlethal
rtTa$^{MCU-/-}$ physiology	1	Mixed (B6 x FVB)	0	Nonlethal
AT2$^{Cx43-/-}$ physiology	1	Mixed (B6 x FVB)	0	Nonlethal
ECSIT$^{+/-}$ physiology	1	B6	0	Nonlethal
rtTA$^{MCU-/-}$ survival	30	Mixed (B6 x FVB)	30	Moderate
ERT2$^{MCU-/-}$ survival	30	B6	30	Moderate
ERT2$^{MCU-/-}$ physiology	30	B6	30	Moderate
pMCUwt survival	10	SW	80	Lethal
BM-MCU survival	10	SW	80	Lethal
AT2$^{CAT+/+}$ survival	50	Mixed (B6 x FVB)	80	Lethal

Supplementary References

1 Luongo, T. S. *et al.* The Mitochondrial Calcium Uniporter Matches Energetic Supply with Cardiac Workload during Stress and Modulates Permeability Transition. *Cell Rep* **12**, 23-34, doi:10.1016/j.celrep.2015.06.017 (2015).

2 Perl, A. K., Wert, S. E., Nagy, A., Lobe, C. G. & Whitsett, J. A. Early restriction of peripheral and proximal cell lineages during formation of the lung. *Proc Natl Acad Sci U S A* **99**, 10482-10487, doi:10.1073/pnas.152238499 (2002).

3 Dai, D. F. *et al.* Mitochondrial oxidative stress mediates angiotensin II-induced cardiac hypertrophy and Galphaq overexpression-induced heart failure. *Circ Res* **108**, 837-846, doi:10.1161/CIRCRESAHA.110.232306 (2011).

4 Luchsinger, L. L., de Almeida, M. J., Corrigan, D. J., Mumau, M. & Snoeck, H. W. Mitofusin 2 maintains haematopoietic stem cells with extensive lymphoid potential. *Nature* **529**, 528-531, doi:10.1038/nature16500 (2016).

5 Ishihara, N. *et al.* Mitochondrial fission factor Drp1 is essential for embryonic development and synapse formation in mice. *Nat Cell Biol* **11**, 958-966, doi:10.1038/ncb1907 (2009).

6 De Stefani, D., Raffaello, A., Teardo, E., Szabo, I. & Rizzuto, R. A forty-kilodalton protein of the inner membrane is the mitochondrial calcium uniporter. *Nature* **476**, 336-340, doi:10.1038/nature10230 (2011).

7 Wert, S. E., Glasser, S. W., Korfhagen, T. R. & Whitsett, J. A. Transcriptional elements from the human SP-C gene direct expression in the primordial respiratory epithelium of transgenic mice. *Dev Biol* **156**, 426-443, doi:10.1006/dbio.1993.1090 (1993).

8 Waypa, G. B. *et al.* Hypoxia triggers subcellular compartmental redox signaling in vascular smooth muscle cells. *Circ Res* **106**, 526-535, doi:10.1161/CIRCRESAHA.109.206334 (2010).

9 Boyden, E. S., Zhang, F., Bamberg, E., Nagel, G. & Deisseroth, K. Millisecond-timescale, genetically targeted optical control of neural activity. *Nat Neurosci* **8**, 1263-1268, doi:10.1038/nn1525 (2005).

10 Smirnova, E., Shurland, D. L., Ryazantsev, S. N. & van der Bliek, A. M. A human dynamin-related protein controls the distribution of mitochondria. *J Cell Biol* **143**, 351-358, doi:10.1083/jcb.143.2.351 (1998).

11 Kelso, G. F. *et al.* Selective targeting of a redox-active ubiquinone to mitochondria within cells: antioxidant and antiapoptotic properties. *J Biol Chem* **276**, 4588-4596, doi:10.1074/jbc.M009093200 (2001).

12 Breslin, J. S. & Weaver, T. E. Binding, uptake, and localization of surfactant protein B in isolated rat alveolar type II cells. *Am J Physiol* **262**, L699-707, doi:10.1152/ajplung.1992.262.6.L699 (1992).

13 Schindelin, J. *et al.* Fiji: an open-source platform for biological-image analysis. *Nat Methods* **9**, 676-682, doi:10.1038/nmeth.2019 (2012).

Supplementary Figure 1

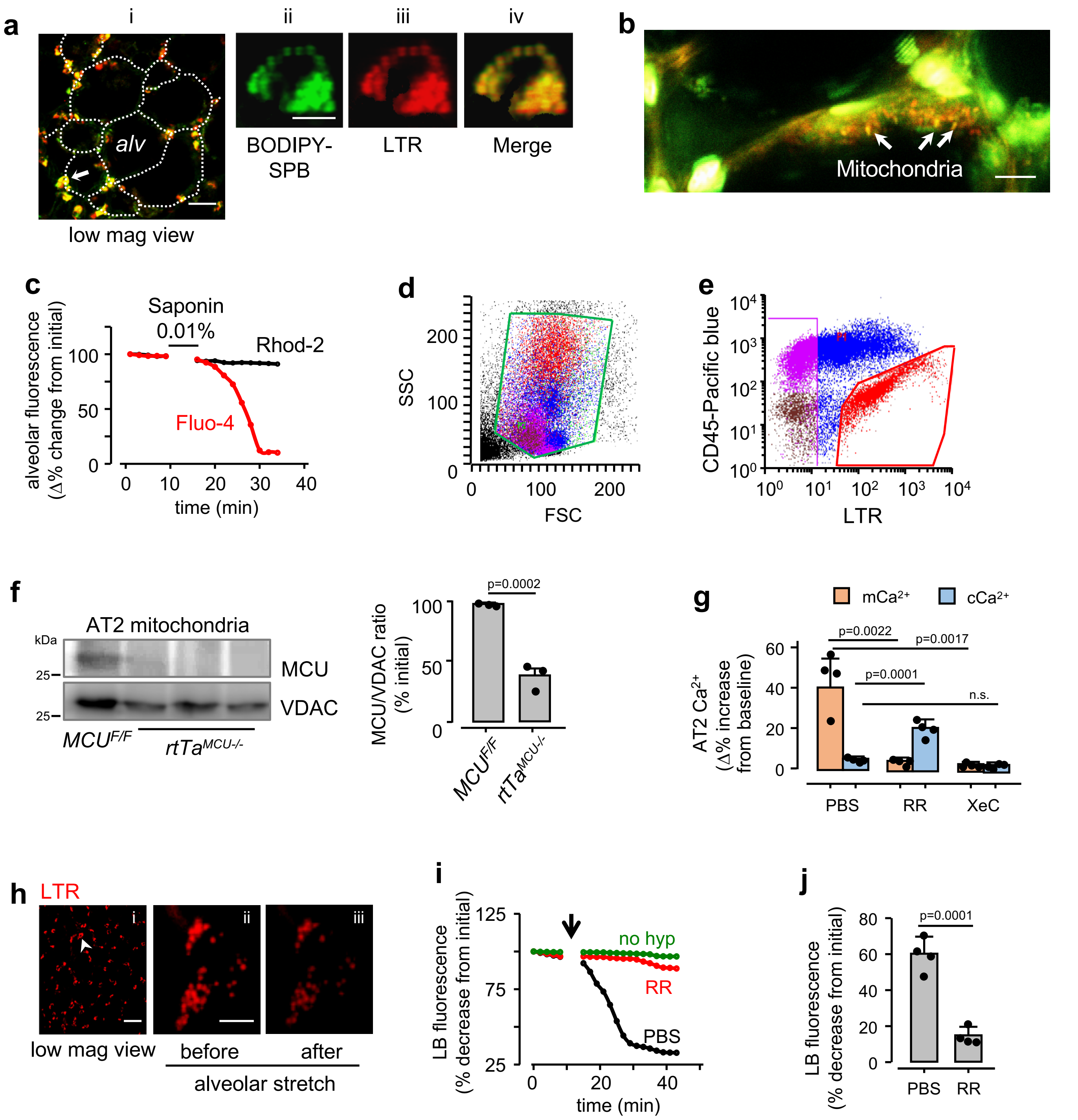

Supplementary Figure 1. Mitochondrial assays in live alveoli. a, Confocal images show AT2 *in situ* at low (i) and high (ii-iv) magnifications for the indicated region (*arrow in i*). Alveoli (*alv*) were stained with the lamellar body localizing dye, lysotracker red (*LTR*) and fluorescent surfactant protein B (*BODIPY-SPB*). Alveolar margins are delineated by dotted lines. Green and red channel renditions show individual lamellar bodies stained with SPB and LTR. Scale bars, 30µm (i) and 5µm (ii-iv). Repeat images were taken at 3 different locations per lung in 4 lungs per group. **b,** Two-photon image (0.5 µm optical section) shows an alveolar septum with rhod-2 loaded mitochondria (arrows, *orange*), fluo-4 loaded cytosol (*green*) and cell nuclei (*white*). Scale bar, 3µm. **c,** Tracings show alveolar microinfusion of saponin selectively decreases the fluorescence of the cytosolic (fluo-4) but not the mitochondrial dye (rhod-2). **d and e**, Flow cytometry plots show the gating strategy for isolating AT2. Cells were gated to exclude debris (*d*). The collected cells (*green box*) were then gated for CD45 and LTR fluorescence (*e*). Cells negative for CD45 and positive for LTR (*red box)* were collected. **f,** We crossed MCU floxed mice with mice expressing an inducible Cre driven by a surfactant protect C promoter (*SPC-rtTa-Cre*). Pre-partum SPC-Cre induction by doxycycline caused epithelial MCU deletion in these mice ($rtTa^{MCU-/-}$), as confirmed by MCU immunoblots in AT2 mitochondria derived from $MCU^{F/F}$ littermates and in alveolar MCU-null ($rtTa^{MCU-/-}$) mice. Group data are quantification of MCU/VDAC band densities. VDAC, voltage dependent anion channel. Bars: mean ± SEM. n=3 mice for each bar, p-value is for two-tailed Student's t-test. **g,** Data are alveolar Ca^{2+} responses to alveolar stretch following indicated treatments. By microinfusion, alveoli were pre-treated with either *PBS*, Ruthenium Red (*RR*) or Xestospongin C (*XeC*). Bars: mean ± SEM. n= 4 lungs each group; groups were compared using one-way ANOVA with Bonferroni correction; *n.s.*, not-significant. **h-j,** Images (*h*), tracings from a single experiment (*i*) and group data (*j*) show determinations of hyperinflation induced surfactant secretion from intact alveoli of live lungs. Confocal image in low magnification (*i*) show an alveolar field stained with lamellar body localizing dye, lysotracker red (*LTR*). Magnification of a select cell (*arrowhead in h*) show a single AT2 (*ii-iii*). Loss of LTR fluorescence following alveolar stretch (*arrow in i*) marks surfactant secretion. Indicated inhibitors were added by alveolar microinfusions. *No hyp*, without alveolar hyperinflation; *RR*, ruthenium red. Scale bars, 10µm (i) and 5µm (ii-iii). Bars: mean ± SEM. n=4 lungs for each bar, p-value is for two-tailed Student's t-test.

Supplementary Figure 2

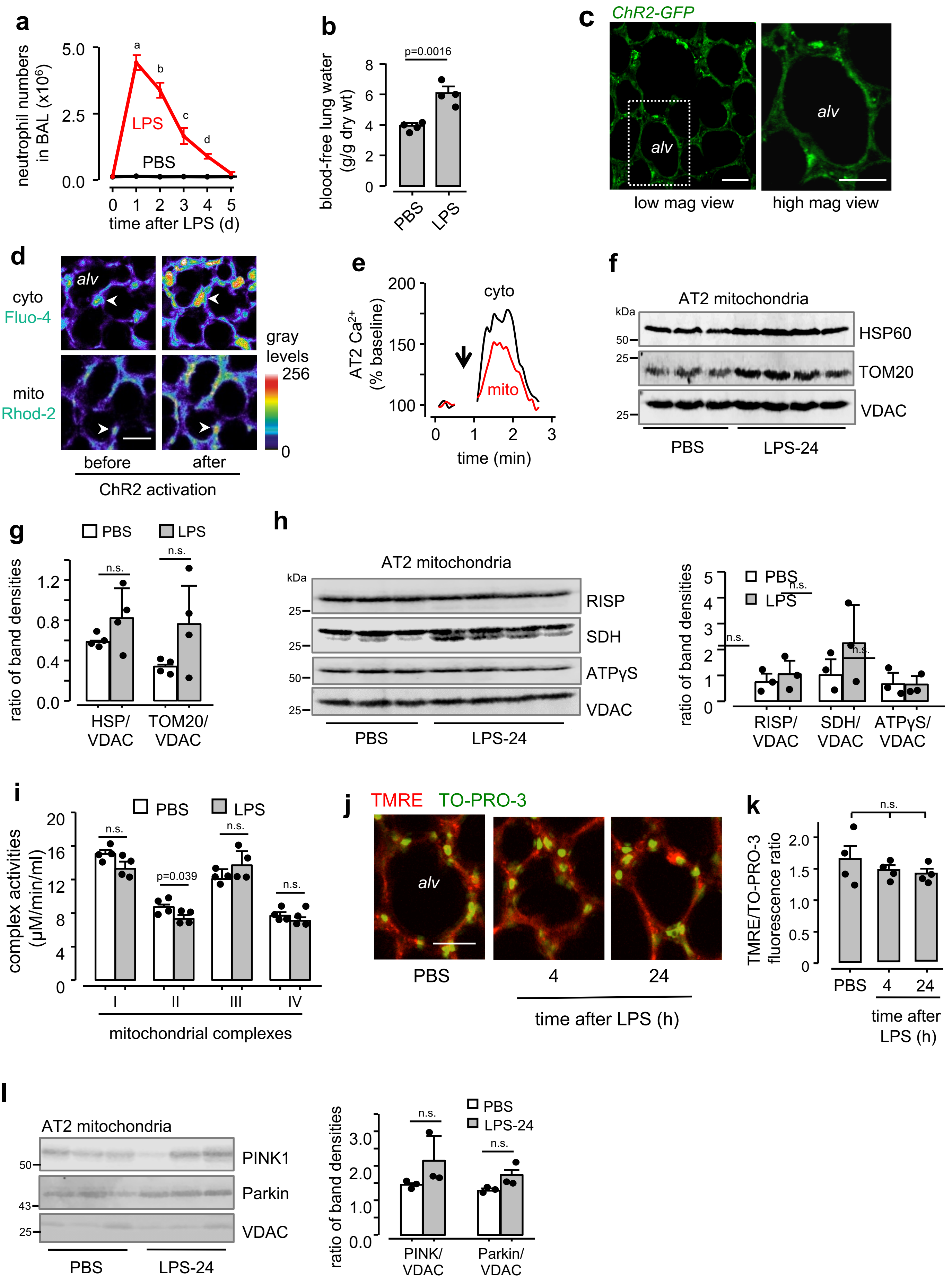

Supplementary Figure 2. LPS depletes MCU. a, Group data are determinations of neutrophil counts in bronchoalveolar lavage at indicated time points following intranasal instillations of LPS at a sublethal dose. Data are mean ± SEM. n=4 mice per group. p-values for time points *a-d* versus the corresponding time points in the PBS group are respectively, 5e-06, 2.5e-05, 0.002, and 0.0001. p-values are for two-tailed Student's t-test. **b,** Determinations of blood-free lung water in mice 24h after indicated instillations. Data are mean ± SEM, n=4 mice per group, p-value is for two-tailed Student's t-test. **c,** Live confocal image in low magnification (*left*) shows GFP fluorescence of the expressed channelrhodopsin (*ChR2*) plasmid in an alveolar (*alv*) field. Magnification of the rectangle shows GFP expression in an alveolus (*right*). *FCK-ChR2-GFP* plasmid was intranasally instilled as a liposomal complex (75µg/mouse) and lungs excised 48h after plasmid instillation. Scale bars, 10µm. Repeat images were taken at 3 different locations per lung in 4 lungs per group. **d and e,** Live confocal images in pseudodocolor rendition (*d*) and tracings from a single experiment (*e*) show simultaneous determination of AT2 (*arrowheads*) cytosolic (*cyto*) and mitochondrial (*mito*) Ca^{2+} responses to a single 10-second ChR2 activation (*arrow in E*). *Alv*, alveolus. Scale bar, 10µm. Repeat images were taken at 3 different locations per lung in 4 lungs per group. **f and g,** Immunoblots for indicated proteins (*f*) and densitometry (*g*) are for AT2 mitochondria derived 24h after intranasal instillations of PBS or LPS at a sublethal dose. Protein densitometry is normalized for VDAC. *HSP60*, heat shock protein 60; *TOM20*, translocase of the outer membrane. Bars: mean ± SEM. n=4 lungs each bar; n.s., not-significant in two-tailed Student's t-test. **h,** Immunoblots and calculated band densities show expression of indicated protein in AT2 mitochondria 24h after intranasal instillations of PBS or sublethal LPS. *RISP*, Rieske's iron-sulfur protein; *SDH*, succinate dehydrogenase; *ATPγS*, adenosine trisphosphate gamma synthase. Bars: mean ± SEM. n= 4 lungs for group; *n*.s., not-significant in two-tailed Student's t-test. **i,** Group data show determination of the activities of electron transfer complexes from alveolar mitochondria 24h after indicated instillations. LPS was instilled at a sublethal dose. Bars: mean ± SEM. n=4 lungs for each bar; p-value is for two-tailed Student's t-test; *n.s.*, not-significant. j **and k,** Images (*j*) and group data (*k*) show alveolar determination of mitochondrial potential. Alveoli (*alv*) were stained with the potentiometric dye, tetramethylrhodamine ethyl ester (*TMRE*) and the nuclear-staining dye, TO-PRO-3. Scale bars, 10µm. Bars: mean ± SEM. n=4 lungs each bar; groups were compared using one-way ANOVA with Bonferroni correction; *n.s.*, not-significant. **l,** In AT2 mitochondria, mitophagy proteins Pink1 and Parkin were immunoblotted and band densities calculated following indicated intranasal instillations. LPS was instilled at a sublethal dose. Bars: mean ± SEM. n= 3 lungs for each bar; *n.s.*, not-significant.

Supplementary Figure 3

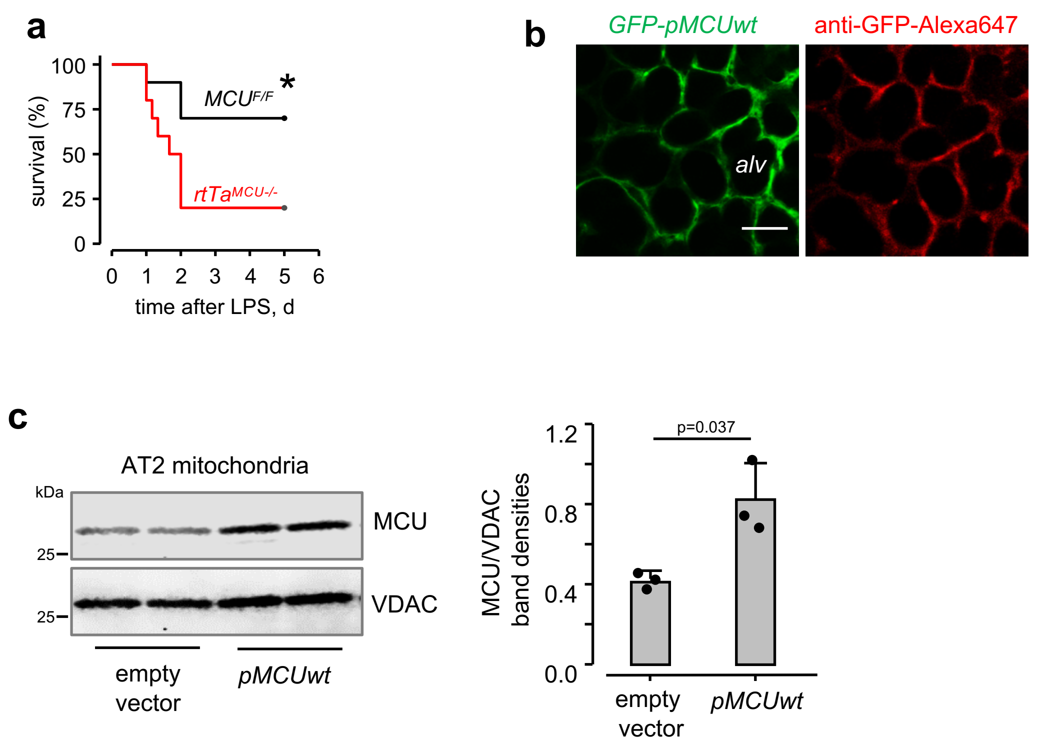

Supplementary Figure 3. MCU overexpression in intact alveoli. **a,** Mouse survival data are for LPS instillations at a moderate dose (30mg/kg). The groups are: *rtTa*$^{MCU-/-}$, mice lacking *MCU* in the alveolar epithelium; *MCU*$^{F/F}$, *MCU*-floxed littermates. n=10 mice in each group, *p=0.022 versus *rtTa*$^{MCU-/-}$ in Log-Rank test. **b,** Images show alveolar (alv) fluorescence of GFP-pMCUwt overexpression (left) and immunofluorescence of the expressed GFP (right). To elicit immunofluorescence, anti GFP mouse Ab tagged with Alexa Fluor-647 (anti-GFP-Alexa647) was microinfused into fixed and permeabilized alveoli. *pMCUwt*, plasmid encoding for full length MCU. Scale bar, 10µm. Repeat images were taken at 3 different locations per lung in 4 lungs per group. **c,** Immunoblots for MCU in AT2 mitochondria derived after intranasal instillations of empty vector and *pMCUwt*. Lungs were excised 48h after intranasal plasmid instillations. Group data are ratios of MCU/VDAC band densities. n=3 lungs each bar. Data are mean ± SEM, p-value is for two-tailed Student's t-test.

Supplementary Figure 4

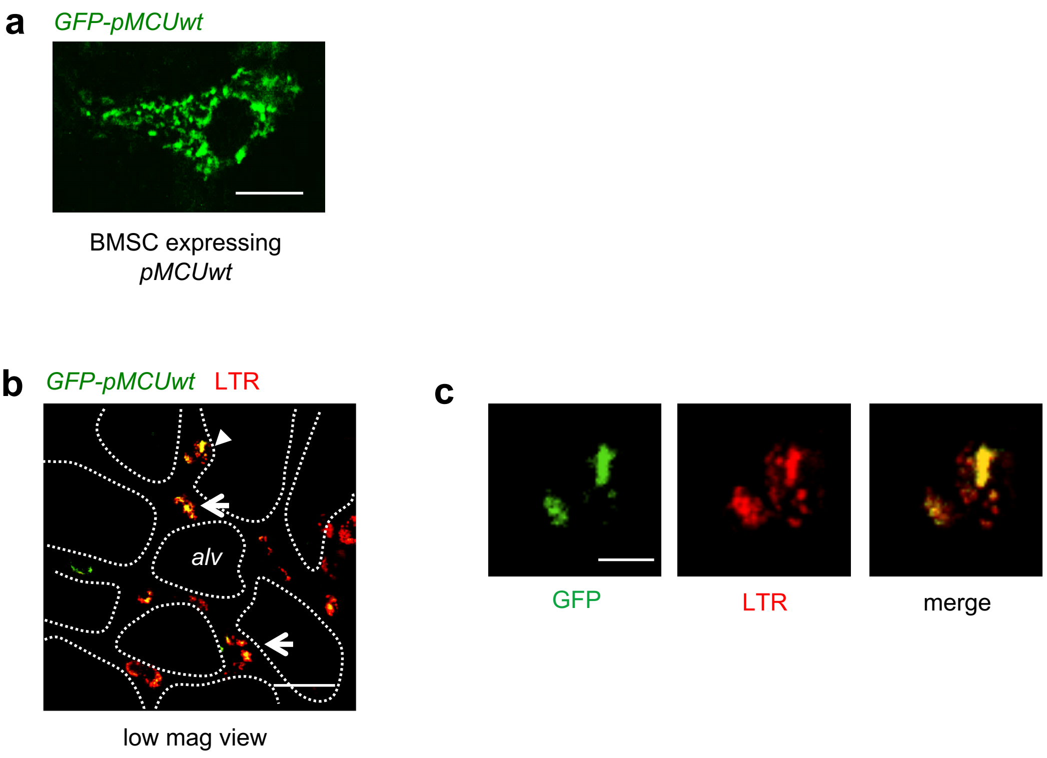

Supplementary Figure 4. BMSCs expressing *pMCUwt* protect against LPS inflammation. **a,** Live confocal image shows a single bone marrow-derived mesenchymal stromal cell (BMSC) expressing GFP-tagged *pMCUwt*. *pMCUwt*, plasmid encoding for full length MCU. Scale bar, 3 µm. Repeat images were taken in 4 independent experiments. **b,** Live confocal image of an LPS-24 lung shows AT2 (LTR, *red*) and instilled BMSCs (arrows and arrowhead) expressing *GFP-pMCUwt* (*green*). Mice were given intranasal LPS instillations at a sublethal dose. BMSCs expressing *pMCUwt* were instilled 4h after LPS instillation. Lungs were excised and imaged 24h after LPS instillation. White lines represent alveolar (*alv*) margin. Scale bar, 15µm. Repeat images were taken at 3 different locations per lung in 4 lungs per group. **c,** Magnified images of a single AT2 (*arrowhead in c*) show in green and red renditions respectively, fluorescence of BMSC-*pMCUwt* (*green*, left) and LTR (*red*, middle). Scale bar, 5µm. Repeat images were taken at 3 different locations per lung in 4 lungs per group

Supplementary Figure 5

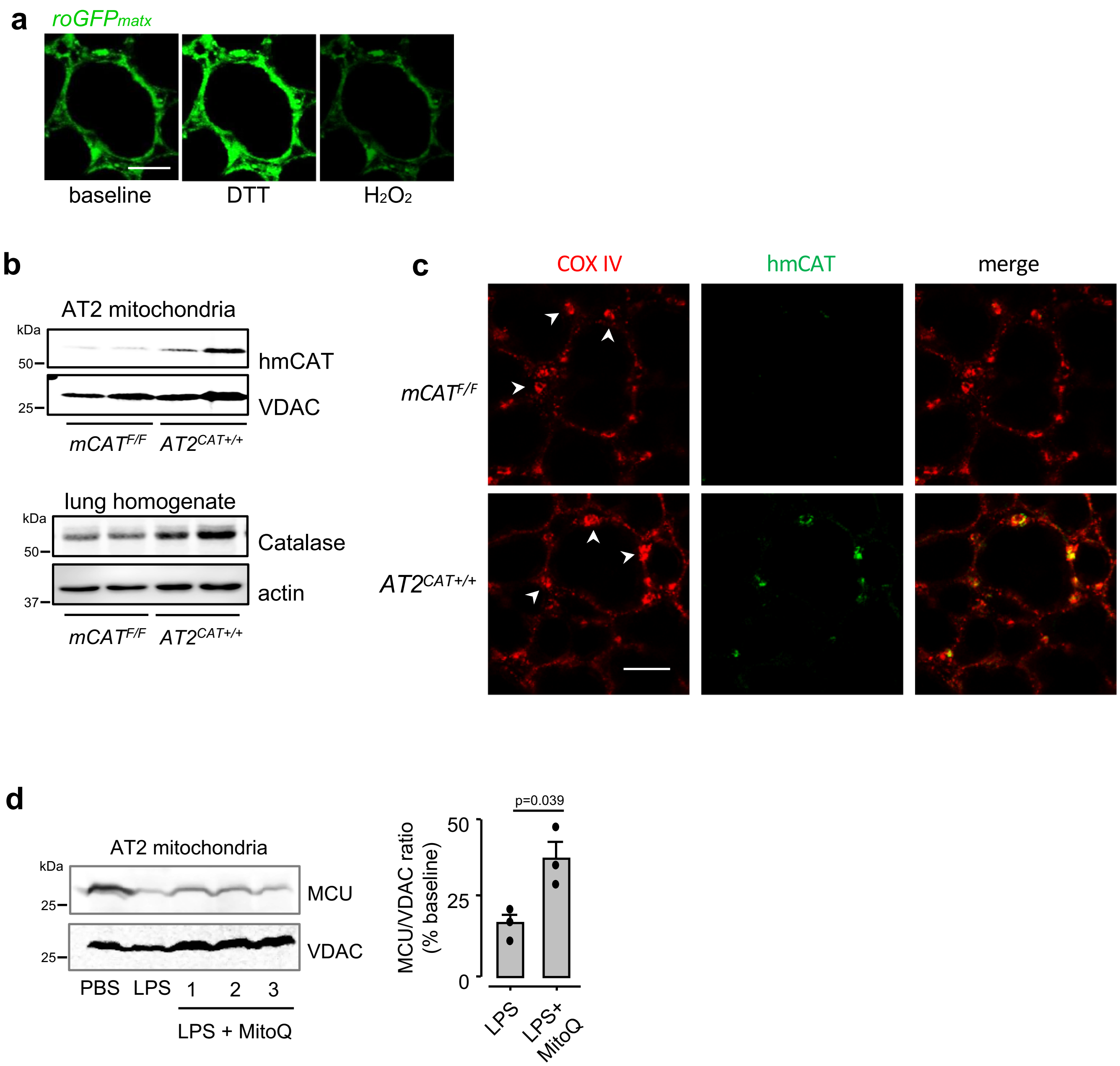

Supplementary Figure 5. Inhibition of mitochondrial H_2O_2 blocks MCU depletion. **a,** Confocal image sequence shows changes in mitochondrial matrix-targeted *roGFP* (*roGFPmatx*) fluorescence following indicated alveolar microinfusions. By alveolar microinfusion, the reducing agent, dithiothreitol (*DTT, 2mM*) and oxidizing agent, hydrogen peroxide (*H_2O_2, 100µM*) were added to obtain respectively, the maximum (fully reduced) and minimum (fully oxidized) fluorescence of the expressed probe. As a metric of mitochondrial H_2O_2, we determined roGFP oxidation (roGFPox) by the relation: roGFPox = (1- [F/F_{DTT}]), where F and F_{DTT} are roGFP fluorescence values for the experiment and after exposure to the reducing agent, DTT. Scale bars, 10µm. Repeat images were taken in 4 independent experiments. **b,** Immunoblots for human catalase (*hmCAT*) in mitochondria derived from AT2 (*upper*) and total catalase in lung homogenates (*lower*). $mCAT^{F/F}$, floxed mice for mitochondrial catalase (mCAT); $AT2^{CAT+/+}$, mice expressing catalase in alveolar mitochondria. n=3 lungs each group. **c,** Images show *in situ* immunofluorescence of human catalase (*hmCAT*, *green*) and cytochrome oxidase IV (*COX IV*, *red*) in AT2 (arrowheads). Scale bar, 10µm. Repeat images were taken in 4 independent experiments. **d,** MCU immunoblots in AT2 mitochondria (*left*) following indicated treatments. Mitochondria-targeted antioxidant MitoQ (mitoquinone mesylate, 100nM) was intranasally instilled 4h after intranasal instillation of LPS at a sublethal dose. Lungs were excised 24h after PBS or LPS instillations. Group data (*right*) are ratio of MCU and VDAC band intensities. Bars: mean±SEM. n= 3 lungs each bar, p-value is for two-tailed Student's t-test.

Supplementary Figure 6

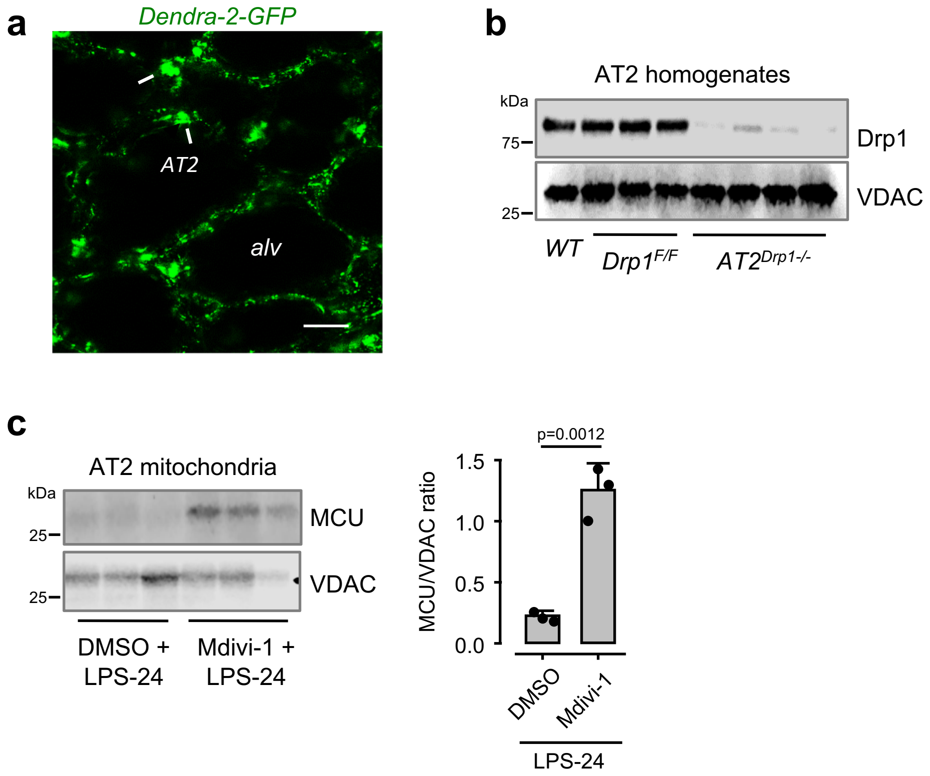

Supplementary Figure 6. LPS causes mitochondrial redistribution. **a,** Live confocal image shows alveolar (*alv*) fluorescence of the mitochondrial matrix-targeted protein, Dendra-2. Mitochondrial Dendra-2 expression was induced by breeding *Pham$^{F/F}$* mice with *E-2A cre* mice. Expression of Dendra-2 is regulated by a cytochrome oxidase 8 (*COX8*) promoter. A high magnification image of a single AT2 is presented in Figure 4A. Scale bar, 5µm. Repeat images were taken in 4 independent experiments. **b,** Drp1 immunoblots in AT2 homogenates derived from wild-type (*WT*), Drp1 floxed mice (*Drp1$^{F/F}$*) or from Drp1 knockout mice in the AT2 (*AT2$^{Drp1-/-}$*). We crossed *Drp1$^{F/F}$* mice with mice expressing an inducible Cre driven by a surfactant protect C promoter (*SPC-rtTa-Cre*). Post-partum SPC-Cre induction by doxycycline caused Drp1 deletion in AT2 (*AT2$^{Drp1-/-}$*). n=3 lungs each group. **c,** MCU immunoblots and densitometry are for freshly isolated AT2 mitochondria following indicated treatments. Wild-type mice were intranasally instilled for 3 days at 12h intervals with 30 mg/kg of the selective Drp1 inhibitor, *Mdivi-1* or vehicle (*DMSO*, dimethyl sulfoxide) followed 24h later by intranasal instillations of LPS at a sublethal dose. Lungs were excised and AT2 mitochondria isolated 24h after LPS instillations. Bars: mean ± SEM. n=3 lungs for each bar. p-value is for two-tailed Student's t-test.

Supplementary Figure 7

a

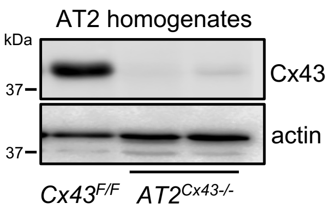

Supplementary Figure 7. Generation of connexin 43 (Cx43) deleted mice in AT2. a, We crossed connexin 43 (Cx43) floxed mice (*Cx43$^{F/F}$*) with mice expressing an inducible Cre driven by a surfactant protect C promoter (*SPC-rtTa-Cre*). Post-partum SPC-Cre induction by doxycycline caused AT2 Cx43 deletion in these mice (*AT2$^{Cx43-/-}$*), as confirmed by Cx43 immunoblots in AT2 homogenates derived from mice of indicated genotype. n=3 lungs each group.
