# Supplementary material for: The mitochondrial calcium uniporter of pulmonary type 2 cells determines severity of acute lung injury
Source: Nat Commun. 2022 Oct 3;13:5837. doi: 10.1038/s41467-022-33543-y (PMC9529882; doi:10.1038/s41467-022-33543-y)
Supplement: Supplementary file 3 — Description of Additional Supplementary Files [file 41467_2022_33543_MOESM3_ESM.pdf]

Description of Additional Supplementary Files

File name: Supplementary Movie 1.

Description: Alveolar stretch causes mCa^{2+} entry.

The movie has three segments. In the first segment, image sequences show baseline fluorescence of a single AT2 (*cuboidal cell in the left*) in a live alveolus stained with the mitochondrial Ca^{2+}-sensing dye, rhod-2. The second segment shows dark images corresponding to the period when alveolar stretch was applied. The third segment shows mages from the post-stretch phase where rhod-2 fluorescence increases denoting increase of mitochondrial Ca^{2+}.
