# Supplementary figures and images for: The mitochondrial calcium uniporter of pulmonary type 2 cells determines severity of acute lung injury
Source: Nat Commun. 2022 Oct 3;13:5837. doi: 10.1038/s41467-022-33543-y (PMC9529882; doi:10.1038/s41467-022-33543-y)

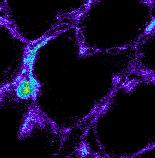

Supplement: Supplementary file 4 — Supplementary Video 1 [file 41467_2022_33543_MOESM4_ESM.tif]
